# Development of an Analytical Model for Predicting the Tensile Modulus of Complex Polypropylene Compounds

**DOI:** 10.3390/polym16233403

**Published:** 2024-12-03

**Authors:** Lukas Seifert, Lisa Leuchtenberger-Engel, Christian Hopmann

**Affiliations:** Institute for Plastics Processing (IKV) in Industry and Craft at RWTH Aachen University, Seffenter Weg 201, 52074 Aachen, Germany

**Keywords:** polypropylene, tensile modulus, analytical model, artificial neural network, polymer blends, compound formulation

## Abstract

The extensive use of polypropylene (PP) in various industries necessitates the development of efficient and reliable methods for predicting the mechanical properties of PP compounds. This study presents the development of an analytical model (AM) designed to predict the tensile modulus for a dataset of 64 PP compounds with various fillers and additives, including chalk, impact strength modifiers, and peroxide additives. The AM, incorporating both logarithmic and linear components, was benchmarked against an artificial neural network (ANN) to evaluate its performance. The results demonstrate that the AM consistently outperforms the ANN, achieving lower mean absolute error (MAE) and higher coefficient of determination (R^2^) values. A maximum R^2^ of 0.98 could be achieved in predicting the tensile modulus. The simplicity and robustness of the AM with its 14 fitting parameters compared to the ~1300 parameters of the ANN make it a useful tool for the plastics industry, providing a practical approach to optimising compound formulations with minimal empirical testing.

## 1. Introduction

Plastics have become an indispensable part of modern life, and are deeply embedded in various industries due to their versatility, durability, and cost-effectiveness. Among the most important sectors for plastic products in Europe are the packaging industry, which accounts for 39% of total plastic use, the construction industry with 21%, and the automotive industry with 9% [1,2]. These industries rely heavily on commodity plastics such as polypropylene (PP) and polyethylene (PE), which together account for almost half of all plastic applications. In the packaging sector in particular, polyolefins such as PP and PE are used predominantly, often alongside polyethylene terephthalate (PET), due to their favourable balance of mechanical properties and processability [1].

One of the key mechanical properties that determines the performance of plastic products is the modulus of elasticity, or the tensile modulus. This property describes the relationship between tensile stress (force per unit area) and strain (proportional change in length) and indicates the stiffness of the material and its resistance to deformation under load. A higher tensile modulus means that the material is stiffer, which is often desirable in applications where structural integrity is critical [3].

In plastic packaging, the tensile modulus is particularly important. It has a direct impact on how well the packaging retains its shape and protects its contents during mechanical stresses such as stacking, transportation, or handling. A packaging material with an appropriate tensile modulus contributes to packaging that is strong enough to prevent deformation and potential damage, yet remains lightweight and cost effective [3]. Achieving this balance is essential to developing packaging solutions that are both durable and functional, meeting the stringent demands of modern logistics and consumer expectations. Examples of such packaging applications include rigid containers, flexible films, and caps or closures, all of which require a careful balance between strength, flexibility, and cost efficiency to meet industry demands. Furthermore, during the packaging process of various consumer goods or foods, the packaging container or film itself must withstand a multitude of forces during its handling and filling.

## 2. Development of New Compound Recipes

The modulus of elasticity of plastics can be finely tuned through formulation development. This involves not only the choice of base polymer, but also systematic modification by blending different polymers, incorporating fillers (such as chalk or talc), or by adding specific additives [4,5,6,7,8,9]. However, modifying these components is not straightforward, as changes intended to improve the tensile modulus can simultaneously affect other critical properties, such as the viscosity of the compound. As a result, the formulation development process is complex and requires careful consideration to achieve the desired balance of properties [5,10,11].

### 2.1. Process of Recipe Development in the Industry

Traditionally, the industry has relied heavily on the expertise of experienced compounders to develop or refine compound formulations. While their knowledge and intuition are invaluable, this empirical and iterative approach is often labour-intensive, time-consuming and lacks reproducibility. This method involves numerous cycles of compounding, testing, and adjusting formulations, which can be resource-intensive, especially when fine-tuning existing formulations or developing new ones to meet specific performance criteria [11,12]. The challenge of predicting material properties when blending different polymers further complicates this process, often requiring extensive trial and error to achieve the desired results [5,10,13].

In recent years, machine learning techniques, particularly artificial neural networks (ANNs), have shown great promise in aiding the development of new compounds. For example, research by Lopez-Garcia et al. has shown that various machine learning models can predict the mechanical properties of fibre-reinforced compounds with high accuracy, achieving model scores (R^2^ values) as high as 0.96, indicating an almost perfect match between the model prediction and the experiment [14]. These models have also proved effective in optimising specific properties such as colour and impact resistance in polyamides [15]. 

The application of machine learning in materials science has expanded rapidly, with studies highlighting its potential to accelerate the discovery and optimisation of materials using large datasets and complex models [11,16,17]. Deepthi et al. have proven that machine learning methods can be used for the optimisation of copper coating processes on graphite powder [18]. Other investigations utilise machine learning for the prediction of the tensile modulus of graphene-reinforced compounds depending on other provided data, such as measurements from dynamic mechanical analysis [19]. 

However, the success of these data-driven approaches is largely dependent on the availability of comprehensive datasets, which must include detailed records of formulation components, processing conditions, and fully characterised material properties [11]. The application of such data-driven methods becomes particularly challenging in environments where documentation is incomplete or where data on new materials or formulations are scarce. While there is ongoing research focused on identifying optimal experimental designs that minimise the effort required to train ANNs and reduce the number of trials required, the need to generate new data for accurate model training remains a significant hurdle in the industry [11]. 

### 2.2. Proposed Aim of This Paper 

As outlined above, the current approach, which is used in many companies to develop new formulations to achieve a defined moduli of elasticity, is largely iterative and unsystematic, often requiring considerable time and resources. To improve the current methodology of developing new or adjusting existing recipes, more systematic methods are being investigated within this paper.

While AI-based methods, such as those using ANNs, offer a more systematic approach when large datasets are available, they are still limited. Even with optimised experimental designs, a significant number of tests must be performed and the ability of AI models to generalise to new formulation components is often limited by the type and amount of data available.

This paper proposes the development of an analytical mathematical model (AM) specifically designed to predict the modulus of elasticity of complex PP formulations. Unlike AI models, which can require extensive data and computational resources, this analytical model can be characterised by only a few modifiable fitting parameters. These parameters can be easily adjusted to accommodate new formulation components, making the model highly adaptable and practical for industrial use. The effectiveness of such models has already been proven in their application on the melt flow rate (MFR) and shear viscosity of PP blends [10].

The primary objective of this work is to demonstrate that such an AM can provide accurate predictions for the tensile modulus of PP compounds across different formulations, with significantly less empirical data required compared to traditional AI methods. To validate the effectiveness of this approach, the paper also includes a comparative analysis with an ANN-based model to evaluate the performance of both models in terms of prediction accuracy depending on the available amount of data. The results of this comparison will highlight the potential advantages and limitations of each approach, providing valuable insights for future applications in polymer formulation development.

## 3. Experimental

In order to obtain experimental data on the tensile modulus, depending on the composition of blends, additives, and fillers, different compound formulations were identified and produced.

### 3.1. Materials and Characterisation

Four virgin homopolymer PP grades were used for the experiments. These were supplied by LyondellBasell (Rotterdam, The Netherlands) and Saudi Basic Industries Corporation (SABIC) (Riyadh, Saudi Arabia). All four polymers selected for this study are commonly used in the manufacturing of packaging applications and differ only in their mechanical and rheological properties. The additives used to specifically adjust the tensile modulus of the compounds produced were a peroxide masterbatch supplied by Polyvel Europe GmbH (Jork, Germany) and an impact modifier masterbatch supplied by DOW Inc. (Midland, MI, USA) [20,21]. Two common types of chalk supplied by OMYA GmbH (Oftringen, Switzerland) were used as fillers. The MFR values and tensile moduli from the data sheets and the designation for the subsequent tests are given in Table 1 [22,23].

### 3.2. Laboratory Equipment for Compounding

All materials were compounded on a co-rotating twin screw extruder (Coperion GmbH, Stuttgart, Germany) with a screw diameter of 26 mm. Two different sets of compound compositions were used for the investigations. In order to investigate the effect of blending two types of homopolymer on the tensile modulus, the composition shown in Table 2 was used to blend PP1350 with PP1600 as well as PP1450 with PP1600. Both series were only used for the analysis of the pure blend models in Section 4.1, without any additives or fillers. For those two series only, the machine temperature was set to 220 °C during processing. For all other compounds, including additives and fillers, a blend composition of PP1500 and PP1600 was used. The blending ratios are given in Table 3. The machine temperature was set to 210 °C. The composition of the screw elements consisted only of the conveying screw elements, with a combination of kneading and mixing elements used at the beginning of the process to plasticise the polymers. The speed of the compounder was kept at 300 min^−1^ for all trials.

The blend composition PP1500–PP1600 was the basis for all tests with fillers and additives. Table 4 shows the different percentages of the different fillers and additives. Only one additive or filler at a time was used for compounding with each of the four base compositions. No compounds containing more than one filler or additive were prepared.

To characterise the tensile modulus, type 1A specimens were produced in accordance with DIN EN ISO 527 on an IntElect 100–250 injection moulding machine from Sumitomo (SHI) Demag Plastics Machinery GmbH (Schwaig, Germany) [24]. The specimens were tested on a Z100 tensile testing machine manufactured by ZwickRoell GmbH & Co. KG, Ulm, Germany. A test speed of 1 mm/min was used to determine the tensile modulus in accordance with DIN EN ISO 527 [24]. For each compound, a minimum of five specimens were tested and used to calculate the average tensile modulus.

## 4. Development of Partial Models for the Prediction of the Tensile Modulus

Subsequently, several partial models were developed to predict the tensile modulus of the different compounds as a function of their formulation. Following the development of a model capable of predicting the tensile modulus of the binary blends, additional models were developed and evaluated for the effect of chalk, the impact modifier, and peroxide.

### 4.1. Development of a Model to Predict the Tensile Modulus of Binary Homo Polymer Blends

**Figure 1:**
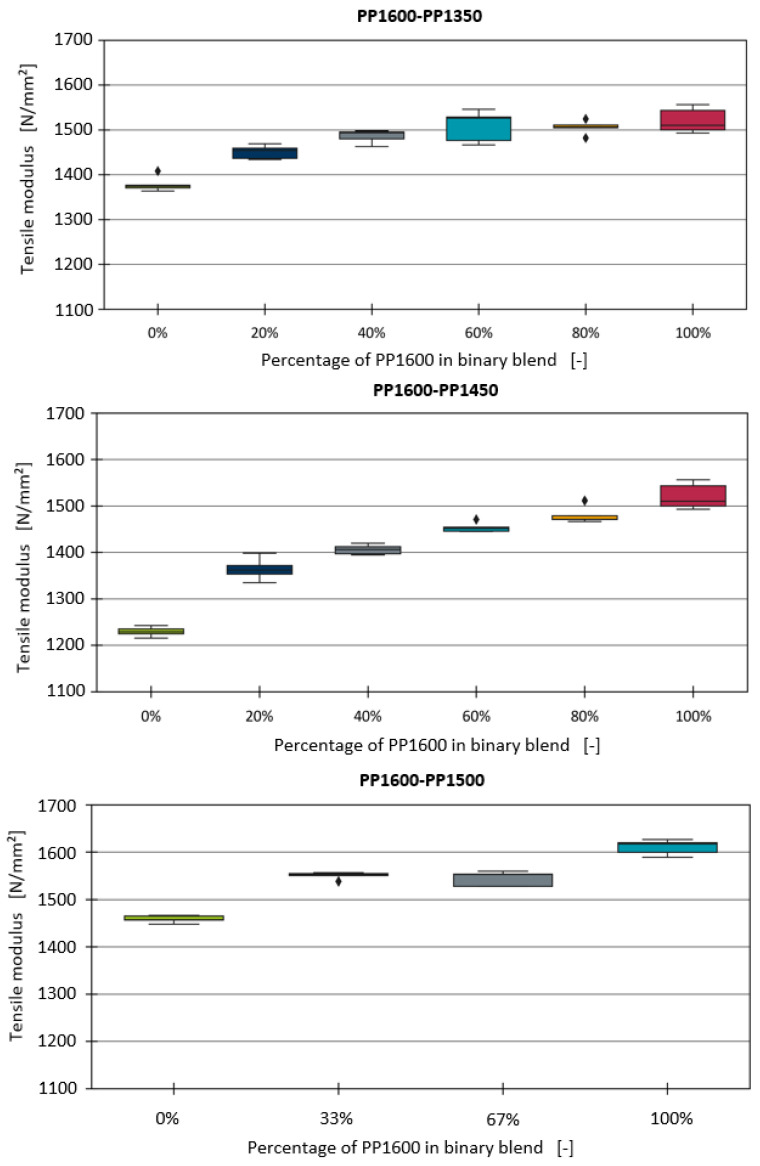
Boxplot diagrams of the tensile moduli of the binary blends.

This section focuses on the development of a predictive model for the tensile modulus of binary homopolymer blends. The tensile modulus measurements for all binary blends, without the inclusion of any fillers or additives, are illustrated in Figure 1.

It was generally observed that as the proportion of PP1600 in the binary blends increases, the tensile modulus of the mixtures also rises, reaching a maximum value with pure PP1600. For the PP1600–PP1350 and PP1600–PP1450 blend series, it is apparent that the relationship between the proportion of PP1600 and the tensile modulus is rather linear when the PP1600 content exceeds 20%. However, the increase in the tensile modulus between 0% and 20% is significantly more pronounced and does not follow a linear trend. Traxler et al. conducted similar investigations on blends of various PP types and investigated mechanical properties, including the tensile modulus. Their findings indicated a similar drop in the tensile modulus at a 20% polymer content, with a higher tensile modulus in homopolymer blends, despite the linear relationship seen in other blend types [5].

To develop a model capable of predicting these relationships, several boundary conditions were established. Firstly, the model should only require the tensile moduli of the pure blend components and their proportions in the blend as input parameters. Additionally, the model should be as simple as possible and applicable across all test series using the same constants. The latter condition is particularly important to ensure the model’s applicability to the materials considered in this series of tests.

Various mathematical functions, including exponential, polynomial, and power functions, were evaluated based on the squared error between each individual function fit and the corresponding measurement data. Ultimately, a logarithmic model was found to best fit the data and minimise the squared error. The logarithmic model is given by Equation (1):

(1) $$E_{mix,\;blend}=\left|E_{A}-E_{B}\right|\times a\times \ln\left(\underset{a}{\max}\left(\frac{E_{A}}{E_{B}},\;\frac{E_{B}}{E_{A}}\right)\times b\times x_{\max\left(E_{A},E_{B}\right)}+\underset{a}{\max}(\frac{E_{A}}{E_{B}},\;\frac{E_{B}}{E_{A}})\times c\right)+\underset{a}{\min}\left(E_{A},E_{B}\right)\times d$$

In this equation, $E_{mix,\;blend}$ represents the tensile modulus of the binary blend, with $E_{A}$ and $E_{B}$ denoting the tensile moduli of the two blend partners. $x_{\max\left(E_{A},E_{B}\right)}$ represents the share of the blend partner with the higher tensile modulus in the binary blend. Due to the logarithmic function’s parameters being dependent on the ratios and differences of the tensile moduli of the two blend partners, this model can be applied across all blends using the same constants. These constants were determined by minimising the error between the model’s predictions and the average tensile moduli of all blends. The fitted constants are provided in Table 5.

**Figure 2:**
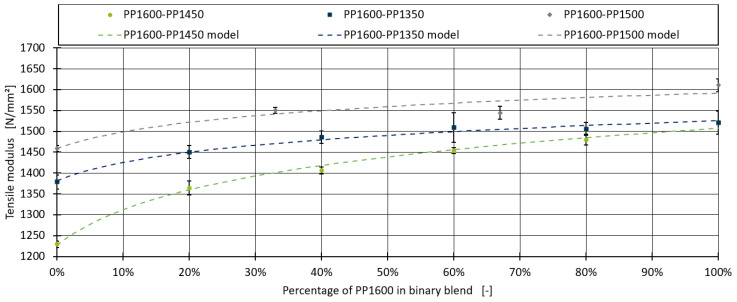
Model predictions and measured data points for the binary blends.

Figure 2 illustrates the application of the model to all three blends. The dotted lines represent the model predictions, while the individual measurements, including their standard deviations, are depicted as dots. The model effectively captures the linear relationship for the higher shares of PP1600 and accurately models the steep increase for shares below 20%.

To quantify the performance of the models, the mean absolute error (MAE) and the coefficient of determination (R^2^) are calculated, with n being the number of samples and E being the tensile moduli of both model predictions and measurements.

(2) $$MAE=\frac{1}{n}\sum_{i=1}^{n}\left|E_{prediction,\;i}-E_{measured,\;i}\right|$$

(3) $$R^{2}=1-\frac{{\left(E_{prediction,\;i}-E_{measured,\;i}\right)}^{2}}{{\left(E_{mean,\;i}-E_{measured,\;i}\right)}^{2}}$$

The R^2^ is a metric used to determine the proportion of variance in a dependent variable that is accounted for by one or more independent variables within a regression model. An R^2^ value of 1 signifies a perfect fit, where the model’s predictions align exactly with the observed data. In contrast, an R^2^ of 0 implies that the model fails to capture any of the variance in the dependent variable. It is important to note that a low R^2^ does not necessarily indicate a poor model; it may reflect significant inherent variability in the data or the challenging nature of modelling in certain domains. Nevertheless, comparing R^2^ values for models applied to the same dataset can reveal important insights into their performance. Additionally, the mean absolute error (MAE) is calculated to assess the model’s prediction accuracy in terms of the average magnitude of errors.

The MAE and R^2^ values of the model, along with the measurement error of the observed data, are summarised in Table 6. For the PP1600–PP1350 and PP1600–PP1450 blends, the MAE is lower than the measurement error, and the R^2^ values are notably high, with 0.984 for PP1600–PP1350 and 0.993 for PP1600–PP1450. However, for the PP1600–PP1500 blend, the MAE of 14.323 exceeds the measurement error of 11.477, and the R^2^ is slightly lower at 0.892. An examination of the PP1600–PP1500 blend series in Figure 2 suggests that the model fits the formulations with 0%, 33%, and 100% PP1600 quite well, while the formulation with 67% PP1600 may be an outlier, as the drop in the tensile modulus between 33% and 100% PP1600 does not align with expectations

### 4.2. Modelling the Tensile Modulus of Blends with Chalk

**Figure 3:**
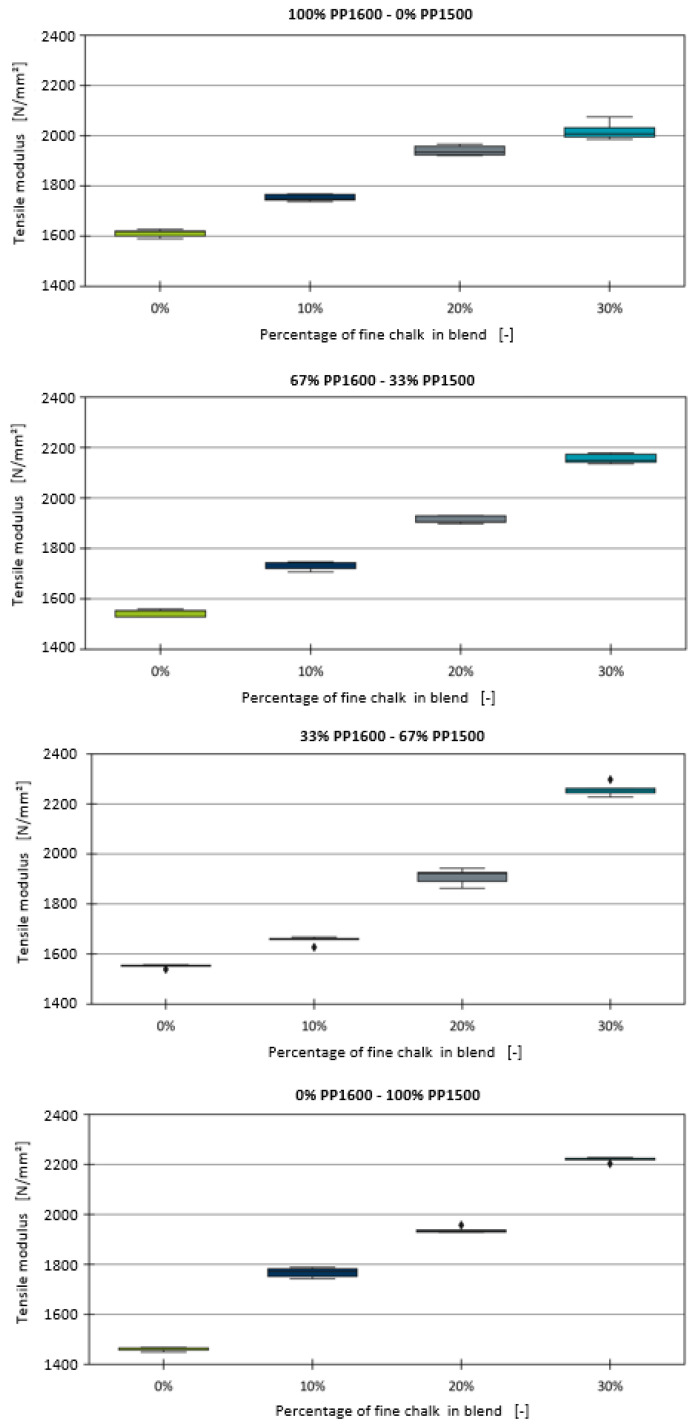
Boxplot diagrams of the tensile moduli of the binary blends with fine chalk.

Following the development of the model for binary homopolymer blends, this section investigates the effect of adding chalk as a filler on the tensile modulus. The boxplot diagrams for the blends containing fine chalk are presented in Figure 3. Both types of chalk used in this study are surface-treated, which inhibits chemical interactions with other components in the formulation. The data clearly show that, for all blends, the tensile modulus increases linearly with the addition of chalk, which is consistent with findings in the literature [6,8].

According to Vollenberg et al., the effect of chalk on the tensile modulus in PP can be described using a modified Kerner equation [9]. However, this equation typically only provides satisfactory results for perfectly adhering chalk and does not account for the particle size of the chalk. Moreover, the equation requires additional information about the tensile modulus of the chalk itself and the Poisson’s ratio of both the PP and the chalk. Such detailed information is often unavailable in technical datasheets, necessitating a more straightforward modelling approach.

To identify a model that is both simple and effective, a linear model following Equation (4) was fitted to the data. For each of the four blends, the tensile modulus $E_{0}$ without any chalk and the slope $m_{Chalk}$ were optimised to minimise the MAE for the individual blends. $x_{Chalk}$ is the share of chalk in the blend.

(4) $$E_{mix,\;chalk}=E_{0}+x_{Chalk}\times m_{Chalk}$$

**Figure 4:**
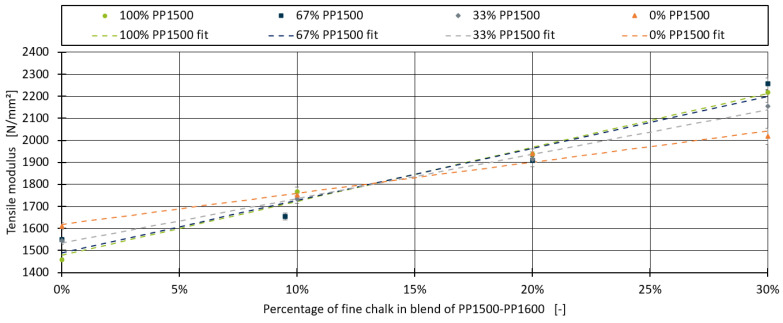
Linear models for the effect of fine chalk, fitted for all four blends.

Figure 4 displays the fitted linear models alongside the average tensile modulus and standard deviation for each blend. The results indicate that the model provides a satisfactory fit to the experimental data. The calculated MAE, R^2^, and measurement error for each blend are summarised in Table 7. For one of the compounds containing 67% PP1500, the target of 10% chalk could not be achieved during the processing of the compounds. Instead, a chalk level of 9.5% was measured during production and used for modelling.

**Figure 5:**
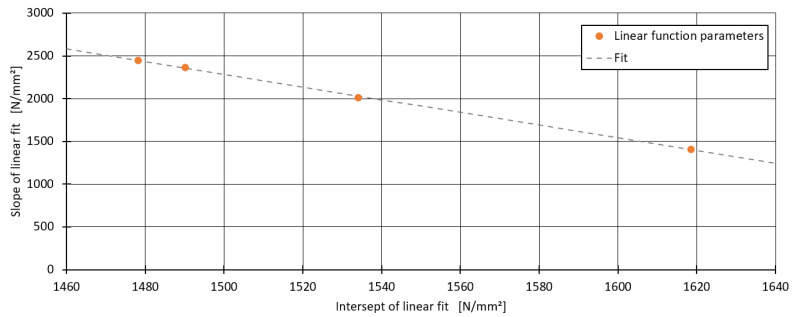
Relationship between the slope of the linear models and $E_{0}$.

For all blends, an R^2^ score of 0.97 or higher was achieved, except for the blend containing 33% PP1600, which included an outlier. For the blends with 67% and 100% PP1600, the MAE was even lower than the measurement error, indicating an excellent fit. Upon further investigation, a linear relationship between the slope of the fitted models and the tensile modulus $E_{0}$ of the base blend without chalk was identified, as shown in Figure 5.

This linear relationship between the slope and $E_{0}$ allows for the development of a unified model for all four blends, as expressed in Equation (5):

(5) $$E_{mix,\;chalk}=E_{0}+x_{Chalk}\times (I_{Chalk}+E_{0}\times s_{Chalk})$$

In this model, instead of determining the slope $m_{Chalk}$ individually for each blend, the parameters were reduced to two: the intercept $I_{Chalk}$ and the slope $s_{Chalk}$ from the linear fit in Figure 5. This approach not only simplifies the model but also enhances its general applicability.

A similar approach was applied to the polymer blends with the second type of chalk. The results for these blends are summarised in Table 8. The measurement error for these blends was similar to that observed for the fine chalk and the MAE was generally lower, with the values of 3.870 for the 100% PP1500 blend and 1.906 for the 33% PP1500 blend being exceptionally low. It is worth noting, however, that only two concentrations of chalk were tested, resulting in three data points for the linear model. Despite this limitation, the model demonstrates good accuracy for both types of chalk, confirming its validity.

### 4.3. Modelling the Tensile Modulus of Blends with Impact Strength Modifier

**Figure 6:**
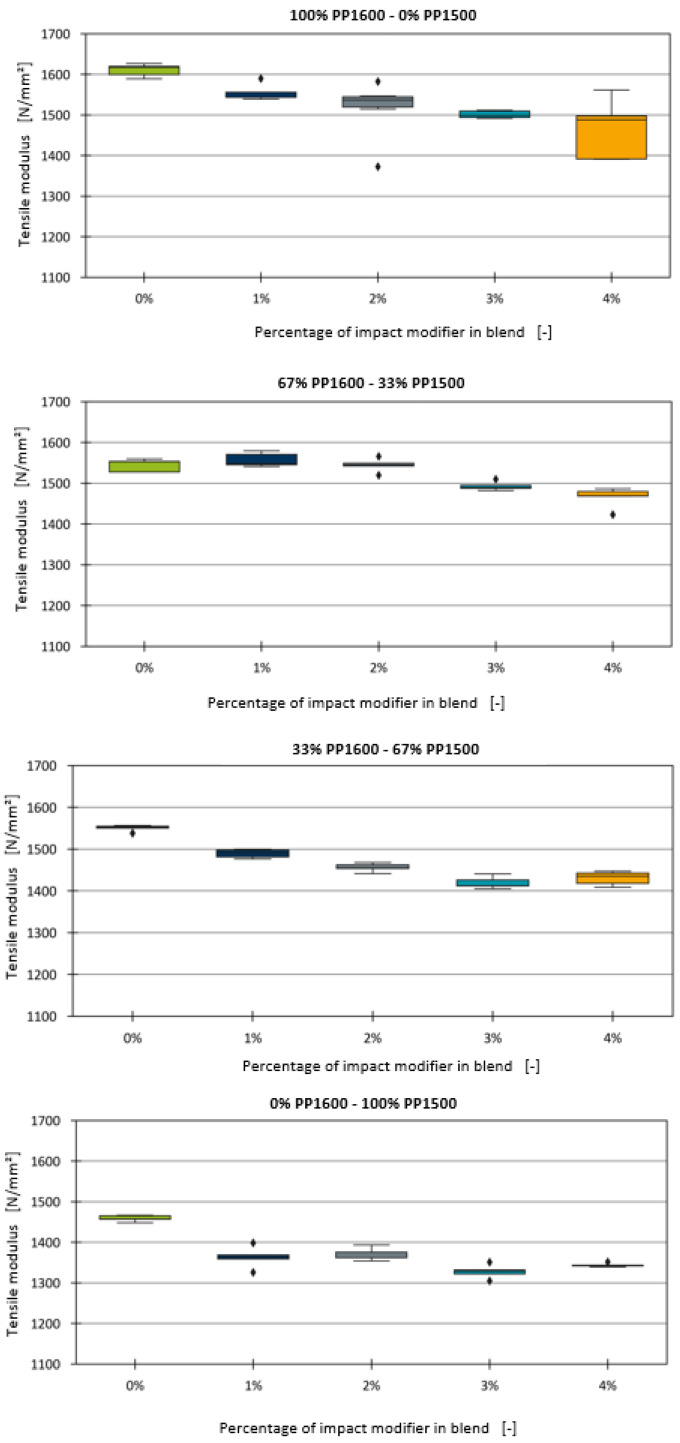
Boxplot diagrams of the tensile moduli of the binary blends with impact strength modifier.

This section addresses the impact of adding an ethylene–octene copolymer as an impact strength modifier on the tensile modulus of the blends. Unlike chalk, which is commonly used in percentages ranging from 10% to 30% in industrial applications, the impact strength modifier is typically used in much smaller amounts, usually less than 5%. Although the primary purpose of this additive is to improve the impact strength of the compound, which is not the focus of this paper, it also has a significant effect on the tensile modulus, which must be considered. The boxplot diagrams for the various blends with the impact strength modifier are shown in Figure 6.

**Figure 7:**
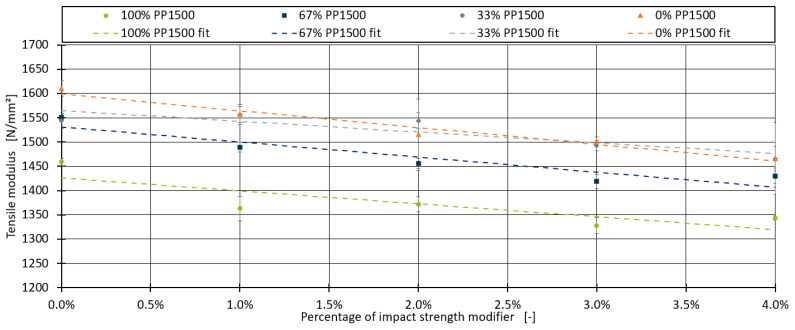
Linear models for the effect of impact strength modifier fitted for all four blends.

Similar to the chalk blends, a linear trend is observed, with the tensile modulus decreasing as the proportion of the impact strength modifier increases. Consequently, a linear model was fitted for each blend, analogously to the approach used for chalk with Equation (4). The fitted linear models, along with the data points and measurement deviations, are depicted in Figure 7.

The linear models generally fit the data well. The MAE and R^2^ values, along with the measurement deviations for each blend, are presented in Table 9.

For the blend series with 100% PP1600, the linear model was highly accurate, achieving an R^2^ value of 0.962 and an MAE of 8.929, which is lower than the measurement error of 38.332. However, for the other blends, the MAE values exceeded the measurement error, indicating less accuracy. One potential reason for these discrepancies, compared to the trials involving chalk, may be the different magnitudes of the component proportions. The chalk trials used percentages ranging from 10% to 30%, where small deviations in the dosing may have had a lesser impact compared to dosing differences in the impact strength modifier, which was used in much smaller amounts (up to 5%). Despite the lower R^2^ values for some blends, the linear models successfully capture the general trend.

**Figure 8:**
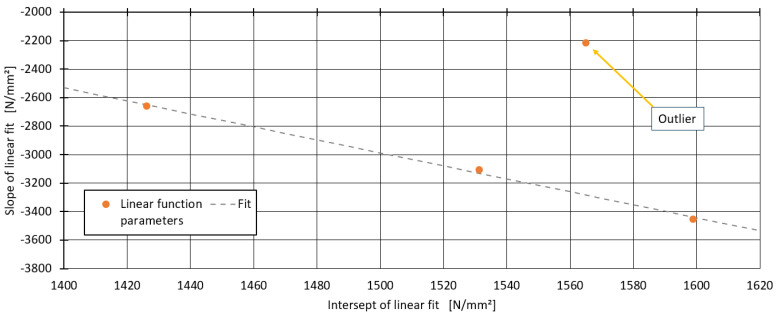
Relationship between the slope of the linear models and ${\;E}_{0}$ for the impact strength modifier.

To further analyse the effect of the impact strength modifier, the relationship between the slope of the linear models and the tensile modulus $E_{0}$ of the pure blends was investigated. This relationship is illustrated in Figure 8. For the blends with 0% PP1600, 33% PP1600, and 100% PP1600, the slopes can be fitted almost perfectly with a linear function. However, the slope for the blend with 67% PP1600 and 33% PP1500 was an outlier and was therefore not considered when determining the generalised model for the impact strength modifier.

### 4.4. Modelling the Tensile Modulus of Blends with Peroxide Additive

**Figure 9:**
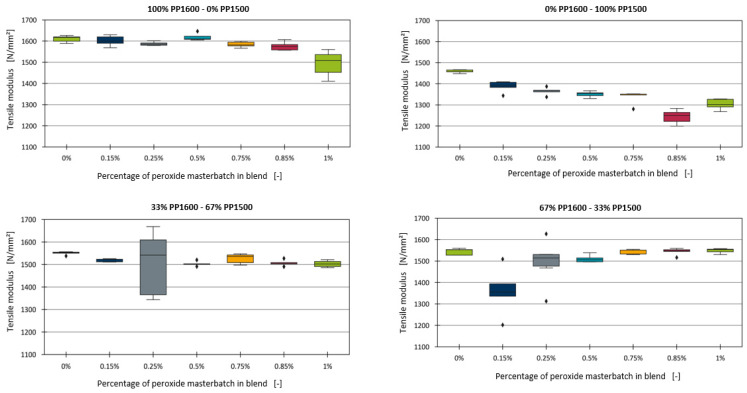
Boxplot diagrams of the tensile moduli of the binary blends with the peroxide-containing additive.

This section explores the effect of adding a peroxide-containing masterbatch on the tensile modulus of the blends. The boxplot diagrams for the different blends with varying percentages of the peroxide additive are presented in Figure 9. For the blends containing 100% PP1600 and 33% PP1600, no significant effect of the peroxide additive on the tensile modulus was observed. A similar lack of effect was noted for the blend with 67% PP1600, with the exception of the formulation containing 0.15% peroxide. However, in the blend with 100% PP1500, a clear decrease in the tensile modulus was identified as the peroxide content increased. 

The differences observed in the effects of peroxide across the blends cannot be fully explained without additional information regarding the formulations of the base materials. Although both types of polypropylene used were homopolymers, differences in the additives, such as stabilisers, present in the virgin compounds may contribute to these discrepancies. Furthermore, a close examination of the data points for the peroxide-containing blends revealed considerable variability in the tensile modulus, particularly in formulations such as 0.25% peroxide for the 33% PP1600 blend, and 0.15% and 0.25% peroxide for the 67% PP1600 blend. 

**Figure 10:**
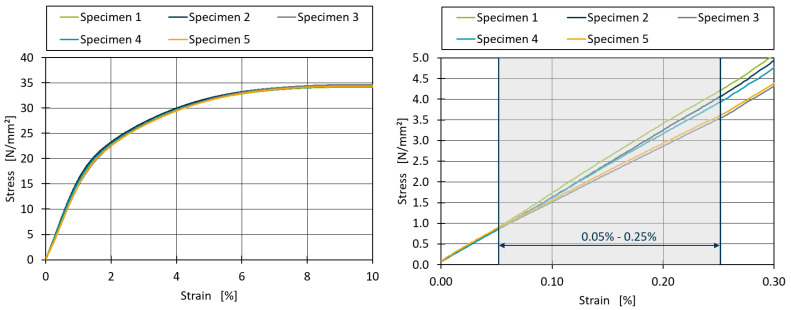
Stress–strain diagram for the blend of 33% PP1600 and 67% PP1500 with 0.25% peroxide additive.

To further investigate this variability, an example of the stress–strain diagram measured during the tensile testing for the 0.25% peroxide formulation in the 33% PP1600 and 67% PP1500 blends is shown in Figure 10. While the stress–strain curves between 0% and 10% strain are relatively consistent for all five specimens tested, significant deviations in the slopes of the curves were observed between 0% and 0.3% strain. According to DIN EN ISO 527, the tensile modulus is calculated as the slope between 0.05% and 0.25% strain [24]. Therefore, the calculated deviation of the tensile modulus for this sample reached values of up to 110 N/mm^2^ compared to the average of 1350 N/mm^2^. These deviations are more pronounced in the trials containing peroxide compared to the other trials, suggesting that the primary purpose of peroxide in the formulations—adjusting polymer viscosity—may influence these results. The injection moulding machine’s parameters were kept constant for all formulations to ensure comparability of the test specimens. However, the introduction of peroxide may have caused inconsistencies during the moulding process, particularly in the filling or holding phases. 

**Figure 11:**
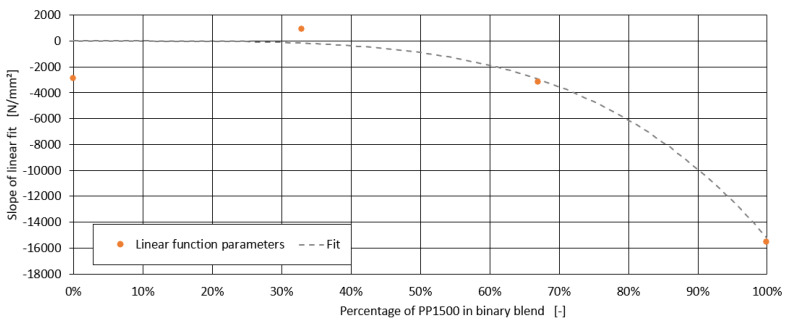
Relationship between the slope of the linear models and the share of PP1500 in the blend.

For further analysis, the three formulations with the highest measurement deviations were excluded. Since a linear relationship can be anticipated in Figure 9, similar to the findings for chalk and the impact strength modifier, a generalised model was sought to describe all four series within a single framework. However, no clear relationship between the slope of the individual linear models and the tensile modulus of the blends without peroxide was found. Instead, a correlation with the proportion of PP1500 in the blend was observed, as shown in Figure 11.

A power model was fitted to these four data points, resulting in the following equation:

(6) $$E_{mix,\;peroxide}=E_{0}+x_{Peroxide}*(p_{1}{*\left(100*x_{PP1500}\right)}^{p_{2}})$$

This equation describes the tensile modulus of the blend series as a function of the peroxide additive proportion $x_{Peroxide}$ and the proportion of PP1500 in the blend $x_{PP1500}$. The fitting parameters for the power model were found to be $p_{1}$ = $-9.63*10^{-5}$ N/mm^2^ and $p_{2}$ = 4.11.

**Figure 12:**
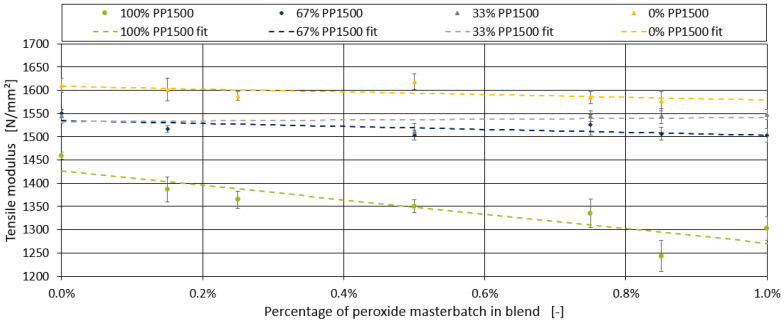
Linear models for the effect of peroxide, fitted for all four blends of PP1500–PP1600.

Figure 12 demonstrates the application of this equation to the four blends. The R^2^ and MAE values, along with the measurement error for each data point, are summarised in Table 10. Although the model provides a reasonably good fit for some blends, the variability in the data indicates that further refinement may be necessary to fully capture the effects of peroxide in these formulations.

## 5. Aggregation of Partial Models to a Complete Model

In the previous sections, individual models were developed to predict the tensile modulus of binary PP blends and the effects of various fillers and additives, such as chalk, impact strength modifiers, and peroxide additives. Each of these models was tailored to specific conditions, focusing on particular blend compositions and additive types. The next logical step is to integrate these partial models into a comprehensive framework that can reliably predict the tensile modulus of a wide range of PP compounds, including those with complex formulations.

To achieve this, an assumption was made that the various fillers and additives do not interact with each other in ways that would significantly alter the underlying relationships identified in the previous models. This assumption allows for the combination of individual models into a single equation that can account for the effects of multiple additives within a single compound. The general form of this aggregated model is given by Equation (7):

(7) $$E_{mix,\;compound}=E_{mix,\;blend}+{\Delta E}_{mix,\;chalk\;fine}+{\Delta E}_{mix,\;chalk\;rough}+{\Delta E}_{mix,impact}+{\Delta E}_{mix,peroxide}$$

In this equation, $E_{mix,\;compound}$ represents the tensile modulus of the complete compound, which includes the effects of blending and the addition of fillers or additives. $E_{mix,\;blend}$ is the tensile modulus of the binary polymer blend without any additives, as previously modelled using a logarithmic function (Equation (8)):

(8) $$E_{mix,\;blend}=\left|E_{A}-E_{B}\right|\times a\times \ln\left(\underset{a}{\max}\left(\frac{E_{A}}{E_{B}},\;\frac{E_{B}}{E_{A}}\right)\times b\times \frac{x_{\max\left(E_{A},E_{B}\right)}}{x_{E_{A}}+x_{E_{B}}}+\underset{a}{\max}(\frac{E_{A}}{E_{B}},\;\frac{E_{B}}{E_{A}})\times c\right)+\underset{a}{\min}\left(E_{A},E_{B}\right)\times d$$

The effects of the individual additives are modelled as adjustments ΔE to this base modulus. For each type of chalk (fine and rough), impact strength modifier, and peroxide, the effect on the tensile modulus is described by equations that are analogous to those developed in earlier sections:

(9) $${\Delta E}_{mix,\;chalk\;fine}=x_{Chalk\_fine}\times (I_{Chalk\_fine}+E_{mix,\;blend}\times s_{Chalk\_fine})$$

(10) $$\;{\Delta E}_{mix,\;chalk\;rough}=x_{Chalk\_rough}\times (I_{Chalk\_rough}+E_{mix,\;blend}\times s_{Chalk\_rough})$$

(11) $${\Delta E}_{mix,impact}=x_{Impact}\times (I_{Impact}+E_{mix,\;blend}\times s_{Impact})$$

(12) $${\Delta E}_{mix,peroxide}=x_{Peroxide}\times (p_{1}{\times \left(100\times x_{PP1500}\right)}^{p_{2}})$$

These equations incorporate the parameters fitted in the previous sections, with $x_{Chalk\_fine}$, $x_{Chalk\_rough}$, $x_{Impact}$, and $x_{Peroxide}$ representing the proportions of the respective additives in the blend. The coefficients I and s correspond to the intercept and slope parameters identified for each additive, while $p_{1}$ and $p_{2}$ are the fitting parameters for the peroxide model.

After developing the complete model by combining these partial models, all parameters were optimised using the full dataset to minimise the overall MAE. The tensile moduli $E_{A}$ and $E_{B}$ of the pure materials were measured, but to ensure the best possible accuracy for the aggregated model, these values were also refined during the optimisation process. The final optimised parameters for the complete model are presented in Table 11.

## 6. Benchmarking with Artificial Neural Networks

After developing and aggregating the partial models into a comprehensive analytical model (AM) for predicting the tensile modulus of polypropylene compounds, the next step is to benchmark this model against an artificial neural network (ANN) approach. This comparison evaluates the effectiveness of the analytical model relative to the ANN in terms of prediction accuracy and robustness, particularly in scenarios where data are limited or noisy.

For the ANN, the Pytorch package (version 2.5.0.) was employed, using Python version 3.10 [25]. A thorough hyperparameter optimisation was performed using the Python package optuna (version 4.1.0) [26]. The ANN architecture (number of layers and neurons per layer), the initialiser and activation functions, as well as the settings for learning rate and weight decay were systematically varied to find the best-suited hyperparameters. All ANNs were trained with a maximum of 1000 epochs and implemented early stopping with a patience of 25 epochs. The optimised hyperparameters for the complete dataset can be seen in Table 12.

The typical approach in machine learning involves splitting the dataset into a training set used to train the model and a test set used to evaluate its performance. For this study, an 80/20 split was chosen, with 80% of the data allocated for training and 20% reserved for testing. To ensure a robust comparison, the dataset was split randomly into training and test sets ten times, mitigating any bias that might arise from a single dataset split. This randomisation helps to prevent scenarios where, for example, all data points for a specific additive end up in the training set and skew the results. Furthermore, for each split into the training and test datasets, the ANN was trained ten times. Similarly, the AM was evaluated on the same splits and fitted ten times as well. Therefore, the resulting values for R^2^ and MAE were calculated on the basis of having 100 trained ANNs and 100 fitted AM.

The analysis was performed on two versions of the dataset: the full dataset, which included all data points (64), and a reduced dataset, from which outliers with a measurement deviation exceeding 50 N/mm^2^ were removed (60 data points). These outliers were all measurements of samples containing a peroxide additive similar to the example discussed in Section 4.4. This allowed for an examination of how well each model handles data variability and outliers.

**Figure 13:**
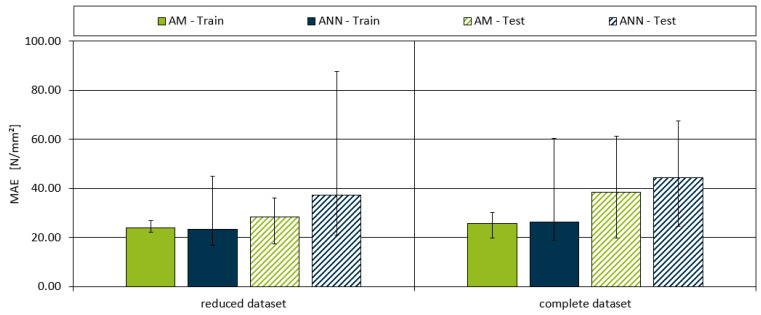
Model MAE for both the aggregated and ANN model for both datasets and the training and testing subsets. The maximum and minimum values of each dataset are indicated by the ends of the error bar.

**Figure 14:**
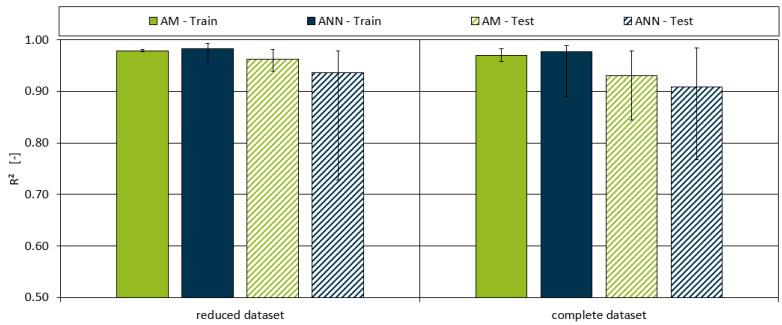
Model R^2^ for both the aggregated and ANN model for both datasets and the training and testing subsets. The maximum and minimum values of each dataset are indicated by the ends of the error bar.

The results of the mean absolute error (MAE) and the coefficient of determination (R^2^) for both models are summarised in Figure 13 and Figure 14. Figure 13 illustrates the MAE values for both the AM and ANN models across all dataset splits, for both training and testing. As expected, the MAE for the test datasets was generally higher than that for the training datasets, reflecting the challenge of generalising the model to unseen data. However, the AM consistently outperformed the ANN, achieving lower MAE values across both the full and reduced datasets. For the full dataset, the AM demonstrated an average MAE of 25.70 for the training data and 38.41 for the test data. In comparison, the ANN produced an average MAE of 26.21 for training and 44.49 for testing. These results indicate that the AM is more accurate overall, with a performance close to the inherent variability in the data, as the measurement error for the full dataset was 24.07. When examining the reduced dataset, which excludes outliers with significant measurement deviations, the AM’s performance improved further.

The AM achieved a minimum MAE of 24.00 for the training data and 28.29 for the test data. In contrast, the ANNs showed only a modest improvement under the same conditions, with average MAE values of 23.23 for training and 37.41 for testing. This suggests that the AM is more robust in handling variability and outliers in the data. When investigating the minimal MAE values throughout all the models, the lowest values of 17.41 for the reduced dataset and 19.78 for the complete dataset were achieved with the AM.

Figure 14 presents the R^2^ values for both models, which indicate the proportion of variance in the dependent variable that is predictable from the independent variables. For the full dataset, the AM achieved an average R^2^ of 0.97 for the training data and 0.93 for the test data, indicating a strong fit to the data. The ANNs’ performance was slightly lower, with R^2^ values of 0.98 for training and 0.91 for testing, further demonstrating that the AM is more effective in capturing the underlying relationships in the data.

## 7. Discussion

A comprehensive analytical model for predicting the tensile modulus of PP compounds has been developed in this paper. The investigated compound formulations consisted of several different types of homopolymer PP, two types of filler chalk, a peroxidic additive, and an impact modifying additive. The analytical model was developed in several steps. A first model was developed to predict the tensile modulus of binary blends using a logarithmic fit, achieving R^2^ values as high as 0.993. Based on this initial model, which is capable of predicting the tensile moduli for the polymer blends, additional analytical models were developed for each filler and additive individually.

For both fillers (rough chalk and fine chalk), linear fitting models were derived and obtained R^2^ values above 0.97. In order to combine the individual linear fitting models for the different polymer blends, a unified model was further developed which captures the relationship between the slope of the linear models and the tensile modulus of the base blend. 

When analysing the impact modifier, a linear relationship similar to the effect of chalk could be deduced, although with lower R^2^ values compared to chalk, particularly for blends with a higher percentage of PP1600. The effect of peroxide additives was more complex, with significant variations in the tensile modulus observed for blends containing 100% PP1500. The performance model developed for peroxide-containing blends showed that although there were some outliers, the general predictive ability of the model remained robust, as indicated by the R^2^ values of around 0.77 for the 0% PP1600 blend.

The individual models for each filler and additive were combined into a single model, described by 14 unique fitting parameters. To evaluate the modelling capability of this analytical approach, a comparison with artificial neural networks was performed. Extensive hyperparameter optimisation was performed to identify the ANN structure best suited to describe the interactions within the provided dataset. For a variety of splits in the original datasets, the ANN was trained on the training dataset, while the AM was fitted to the training dataset. Both models were then evaluated on the individual test datasets.

Comparison of the AM and ANNs showed that the AM achieved a lower average MAE of 25.70 for the training data and 38.41 for the test data from the full dataset, compared to 26.21 and 44.49 for the ANNs. The R^2^ values of the aggregated model also surpassed those of the ANNs, especially in the reduced dataset, with values reaching 0.98 for training and 0.96 for testing. These results underline the superior performance of the AM, considering that it is able to capture the complex interactions of the fillers, additives, and polymers with 14 fitting parameters compared to the ~1300 parameters of the ANNs. Due to the small number of parameters required, it may be possible to transfer the AM to a different twin screw extruder with a different throughput or dimension (e.g., 150 kg/h instead of the 15 kg/h used on the laboratory twin screw extruder in this paper) with only a few trials. 

In addition, the AM is easily adaptable to new additives or fillers. In the simple architecture of the aggregated model, a new recipe component can be modelled in a similar way and simply added to the other components of the recipe. This is not as easy with ANNs due to the need to change their architecture.

Overall, the developed model not only provides a highly accurate and efficient method of predicting the tensile moduli of PP compounds, but also offers a practical tool for industry to optimise formulations with minimal empirical testing. In the development of the current model, the possible side effects of combining several additives that interact with each other were neglected and will be investigated. Future work will explore the extension of this model to other types of polymer and more complex formulations to further improve its applicability and robustness.

## Figures and Tables

**Table 1:** Materials used in the investigation [22,23].

Designation	Product Name	MFR [g/10 min]	Tensile Modulus [MPa]
PP1500	505P	2.0	1500
PP1450	HP525J	3.0	1450
PP1350	HP501M	7.5	1350
PP1600	HP548R	23.0	1600

**Table 2:** Blend composition to investigate the blending of virgin materials without any additives or fillers for the blends PP1350–PP1600 and PP1450–PP1600.

**X_1_**	0%	20%	40%	60%	80%	100%
**X_2_**	100%	80%	60%	40%	20%	0%

**Table 3:** Base composition for the trials including additives and fillers with PP1500–PP1600.

**PP1500**	0%	33%	67%	100%
**PP1600**	100%	67%	33%	0%

**Table 4:** Blend, filler, and additive compositions for the blend of PP1500–PP1600.

Designation	Material	Percentages							
Fine chalk	Omyalite 95 T	10%			20%		30%		
Rough chalk	Omyalite 50 H	10%				20%			
Impact modifier	Engage 8200	1%		2%		3%		4%	
Peroxide additive	Polyvel CR5P	0.15%	0.25%		0.5%	0.75%	0.85%		1.0%

**Table 5:** Constants found for Equation (1) to minimise the squared error between the prediction and the experimental observation.

Constant	a	b	c	d
Value	0.38364	7.52671	0.65223	1.01744

**Table 6:** Evaluation of the model metrics for the binary homo polymer blends.

Blend	Measurement Error	MAE	R^2^
PP1600–PP1350	20.865	5.529	0.984
PP1600–PP1450	13.347	6.628	0.993
PP1600–PP1500	11.477	14.323	0.892

**Table 7:** Evaluation of the model metrics for blends containing fine chalk.

Polymer Ratio	Measurement Error	MAE	R^2^
0% PP1600–100% PP1500	12.058	25.120	0.984
33% PP1600–67% PP1500	19.495	58.256	0.906
67% PP1600–33% PP1500	16.975	13.678	0.993
100% PP1600–0% PP1500	21.436	19.571	0.976

**Table 8:** Evaluation of the model metrics for blends containing rough chalk using Equation (5).

Polymer Ratio	Measurement Error	MAE	R^2^
0% PP1600–100% PP1500	12.711	3.870	0.999
33% PP1600–67% PP1500	13.856	21.500	0.946
67% PP1600–33% PP1500	15.356	1.906	0.999
100% PP1600–0% PP1500	16.017	29.481	0.905

**Table 9:** Evaluation of the model metrics for the prediction of the tensile modulus of the blends containing impact strength modifier.

Polymer Ratio	Measurement Error	MAE	R^2^
0% PP1600–100% PP1500	13.940	22.582	0.680
33% PP1600–67% PP1500	11.648	17.085	0.860
67% PP1600–33% PP1500	16.842	18.422	0.565
100% PP1600–0% PP1500	38.332	8.929	0.962

**Table 10:** Evaluation of the model metrics for blends containing peroxide additive.

Polymer Ratio	Measurement Error	MAE	R^2^
0% PP1600–100% PP1500	22.239	25.814	0.774
33% PP1600–67% PP1500	12.471	10.410	0.473
67% PP1600–33% PP1500	14.366	11.134	0.020
100% PP1600–0% PP1500	16.674	13.862	0.555

**Table 11:** Optimised parameters for minimising the total MAE of the complete model.

Parameter	Value	Parameter	Value
a	0.38364	$I_{Chalk\_fine}$	13,415.550
b	7.52671	$I_{Chalk\_rough}$	8628.090
c	0.65223	$I_{Impact}$	3852.150
d	1.01744	$s_{Chalk\_fine}$	−7.421
$E_{A}$	1458.86	$s_{Chalk\_rough}$	−4.505
$E_{B}$	1610.54	$s_{Impact}$	−4.560
$p_{1}$	−0.0000963	$p_{2}$	4.100

**Table 12:** Optimised hyperparameters for the ANNs.

Parameter	Value/Setting
Layers	2
Neurons layer 1	27
Neurons layer 2	39
Activation function	Relu
Initialisation	Xavier_normal
Learning_rate	0.0006
Weight_decay	0.0073
Batch size	8

## Data Availability

The original contributions presented in this study are included in the article. Further inquiries can be directed to the corresponding author.
